# Alteration of Membrane Fluidity or Phospholipid Composition Perturbs Rotation of MreB Complexes in *Escherichia coli*

**DOI:** 10.3389/fmolb.2020.582660

**Published:** 2020-11-24

**Authors:** Keisuke Kurita, Fumiya Kato, Daisuke Shiomi

**Affiliations:** Department of Life Science, College of Science, Rikkyo University, Tokyo, Japan

**Keywords:** bacterial actin, peptidoglycan, anionic phospholipids, membrane fluidity, cell shape

## Abstract

Gram-negative bacteria such as *Escherichia coli* are surrounded by inner and outer membranes and peptidoglycan in between, protecting the cells from turgor pressure and maintaining cell shape. The Rod complex, which synthesizes peptidoglycan, is composed of various proteins such as a cytoplasmic protein MreB, a transmembrane protein RodZ, and a transpeptidase PBP2. The Rod complex is a highly motile complex that rotates around the long axis of a cell. Previously, we had reported that anionic phospholipids (aPLs; phosphatidylglycerol and cardiolipin) play a role in the localization of MreB. In this study, we identified that cells lacking aPLs slow down Rod complex movement. We also found that at higher temperatures, the speed of movement increased in cells lacking aPLs, suggesting that membrane fluidity is important for movement. Consistent with this idea, Rod complex motion was reduced, and complex formation was disturbed in the cells depleted of FabA or FabB, which are essential for unsaturated fatty acid synthesis. These cells also showed abnormal morphology. Therefore, membrane fluidity is important for maintaining cell shape through the regulation of Rod complex formation and motility.

## Introduction

Bacterial cells maintain their own shape such as rod and sphere (Young, 2003). Most bacterial cells are surrounded by peptidoglycan, a macromolecule composed of glycan strands crosslinked by short peptides, which forms the cell wall. Peptidoglycan can be purified, and electron microscopic observation of the purified peptidoglycan indicated that it determines the bacterial shape (de Pedro et al., 1997). Thus, bacterial cells have to correctly synthesize peptidoglycan to maintain cell shape. In some rod-shaped bacteria, peptidoglycan is synthesized by a supramolecular complex called Rod complex or elongasome, which contains various proteins such as a scaffold protein MreB actin, a membrane protein RodZ, a transglycosylase RodA and a transpeptidase PBP2 (den Blaauwen et al., 2008). In the rod-shaped bacterium *Escherichia coli*, peptidoglycan is synthesized at the central cylinder but not at the cell poles (de Pedro et al., 1997). This localized peptidoglycan synthesis is achieved by limited localization of Rod complex at the central cylinder (Ursell et al., 2014; Kawazura et al., 2017).

MreB is structurally and biochemically homologous to actin (van den Ent et al., 2001, 2014) and localizes at the central cylinder in *E. coli* (Ursell et al., 2014; Kawazura et al., 2017). This localization is important for determination of cell shape and synthesis of peptidoglycan because *E. coli* cells become spherical if they lack MreB (Wachi et al., 1987; Doi et al., 1988; Bendezú and de Boer, 2008) or are treated with A22, which inhibits the assembly of MreB (Iwai et al., 2002; Kawazura et al., 2017). Rod complex or MreB rotates around the long axis of the cell and this rotational motion is coupled with peptidoglycan synthesis (Domínguez-Escobar et al., 2011; Garner et al., 2011 van Teeffelen et al., 2011).

We have shown previously that anionic phospholipids (aPLs: phosphatidylglycerol and cardiolipin), which localize at the cell poles (Mileykovskaya and Dowhan, 2000; Oliver et al., 2014), exclude MreB from cell poles in *E. coli*. Specifically, in the cells lacking aPLs (ΔaPLs), MreB localizes to cell poles as well as to the central cylinder (Kawazura et al., 2017). MreB directly binds to phospholipids through its N-terminal amphipathic helix (Salje et al., 2011). We identified that aPLs could not bind to the assembled form of MreB in the presence of ATP but could bind to a disassembled form of MreB in the absence of ATP (Kawazura et al., 2017). However, detailed molecular mechanism of interaction between MreB and aPLs is still unclear. Thus, the composition of phospholipids affects the localization of MreB (Rod complex), which in turn affects peptidoglycan synthesis (Kawazura et al., 2017). It is known that composition of phospholipids in *E. coli* affects the membrane fluidity (Pluschke and Overath, 1981; Nenninger et al., 2014), which is modulated by the amount of unsaturated fatty acids (Marr and Ingraham, 1962). Interestingly, MreB promotes membrane fluidity and affects membrane protein localization (Strahl et al., 2014). Therefore, we hypothesized that membrane fluidity also affects MreB motion.

In this study, we examined the effects of aPLs on the rotational motion of MreB and identified that aPLs affect not only the localization but also the motion of MreB. We then examined the effect of membrane fluidity on rotational motion of MreB by depleting FabA or FabB protein. When enzymes required for synthesis of unsaturated fatty acids were depleted (encoded by *fabA* or *fabB*), rotational speed of MreB was decreased, suggesting that membrane fluidity affects the rotational speed of MreB.

## Methods

### Bacterial Strains and Growth Medium

All strains were derivatives of *E. coli* K-12 and are listed in Supplementary Table 1. MG1655 is a wild-type (WT) strain. Cells were grown in L broth (1% bacto-tryptone, 0.5% yeast extract, 0.5% NaCl) or M9 medium (0.6% Na_2_HPO_4_, 0.3% K_2_HPO_4_, 0.05% NaCl, 0.1% NH_4_Cl, 0.1 mM MgSO_4_·7H_2_O) containing 0.25% glucose at 37°C. Kanamycin (Kan; 50 μg/mL), ampicillin (Amp; 100 μg/mL), and chloramphenicol (Cm; 20 μg/mL) were added to the culture medium when necessary.

### Strain and Plasmid Constructions

The primers used for strain and plasmid constructions and the plasmids used in this study are listed in Supplementary Tables 2, 3. Detailed methods for strain construction were described in Supplementary Materials.

### Microscopic Observation

Cells were grown to log phase and mounted on a 2% agarose in M9 medium. The cells were observed using an Axio Observer (Zeiss, Oberkochen, Germany), and images were processed using ZEN (Zeiss), Photoshop 2020 (Adobe), and ImageJ. The objective lens was heated by a lens heater (Tokai Hit, Shizuoka, Japan) when necessary. All experiments were repeated two or more times on different days. Analyses of the rotational speed of MreB and RodZ were performed as previously described (Kurita et al., 2019).

### Bacterial Two-Hybrid Assays

Bacterial two-hybrid assays were performed as described previously (Shiomi and Margolin, 2007).

### SDS-PAGE and Immunoblotting

Cells were grown to log phase and samples were subjected to SDS-polyacrylamide gel electrophoresis and immunoblotting using anti-DDDDK antibody (MBL, Nagoya).

## Results

### MreB Motion in Cells Lacking aPLs

**Figure 1:**
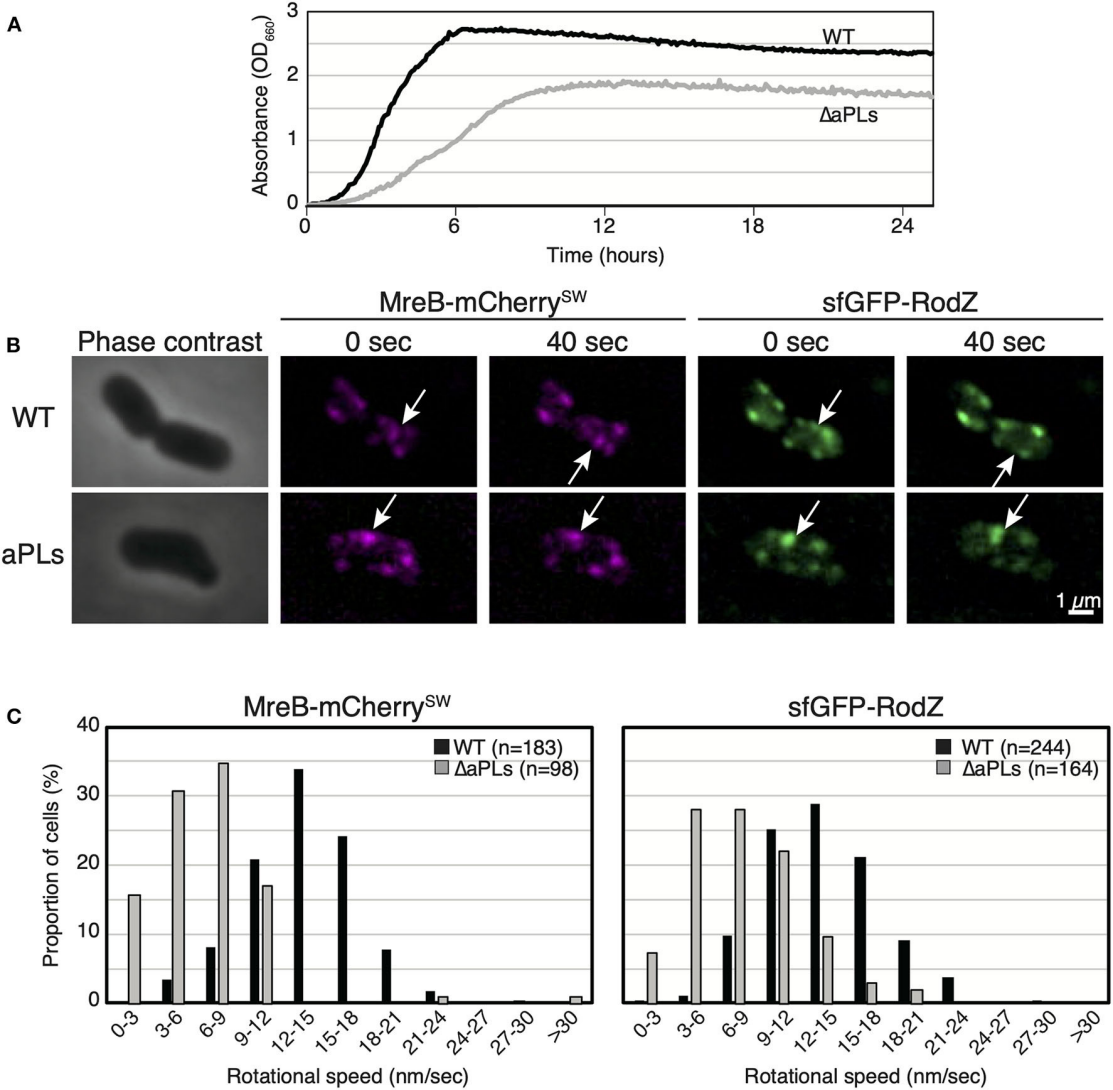
Motion of MreB and RodZ in cells lacking anionic phospholipids (aPLs). **(A)** Growth curves of WT (solid lines) and ΔaPLs (dotted lines) at 37°C. **(B)** Time-lapse images of wild-type (WT) or ΔaPLs cells. Images were taken every 10 s on an M9 agarose pad. Phase-contrast images and fluorescent images are shown. Scale bar: 1 μm. **(C)** Histograms of rotational speeds (nm/s) of MreB and RodZ in WT and ΔaPLs cells. Black and gray bars indicate WT and ΔaPLs, respectively.

We have previously reported that aPLs affect the subcellular localization of MreB (Kawazura et al., 2017). However, it is unknown whether aPLs affect the motion of MreB and thus, that of the Rod complex. Before we analyzed MreB rotation in ΔaPLs cells, we examined the growth of WT (RU1184) and ΔaPLs (RU1185) cells at 37°C. ΔaPLs cells (shown by a gray line) (doubling time: 144.4 ± 3.8 min, mean ± SD) grew significantly slower than WT cells (shown by a black line) (doubling time: 28.8 ± 2.4 min) (Figure 1A), indicating that the growth rate of ΔaPLs cells is slower than that of WT cells. We hypothesized that the slower growth rate correlated with slower peptidoglycan synthesis, which should be reflected in slower MreB and RodZ movement. To analyze the motion of MreB and RodZ, we simultaneously observed the motion of MreB-mCherry^SW^ and sfGFP (super-folder green fluorescent protein)-RodZ in WT (RU1184) and ΔaPLs cells (RU1185) (Figures 1B,C, Supplementary Movies 1A–D, and Supplementary Table 4). In WT cells (RU1184), MreB (14.0 ± 3.8 nm/s) and RodZ (14.1 ± 4.0 nm/s) were simultaneously rotated around the long axis, whereas in ΔaPLs cells (RU1185), the rotational speeds of both MreB (7.1 ± 4.1 nm/s) and RodZ (8.5 ± 3.8 nm/s) were clearly decreased compared to those in WT cells, suggesting that aPLs affect the motion of MreB and RodZ proteins. It is unclear whether different compositions of phospholipids or other factors, such as improper interaction between MreB and RodZ, reduced peptidoglycan synthesis, or reduced membrane fluidity, affected MreB and RodZ motion according to this result. We showed that the interaction between MreB and RodZ in ΔaPLs cells was comparable to that in WT cells (Supplementary Figure 1), suggesting that the decreased motion of MreB and RodZ in ΔaPLs cells may at least not be due to a lack of proper interaction between MreB and RodZ.

### MreB Motion in Cells Lacking aPLs at Higher Temperatures

**Figure 2:**
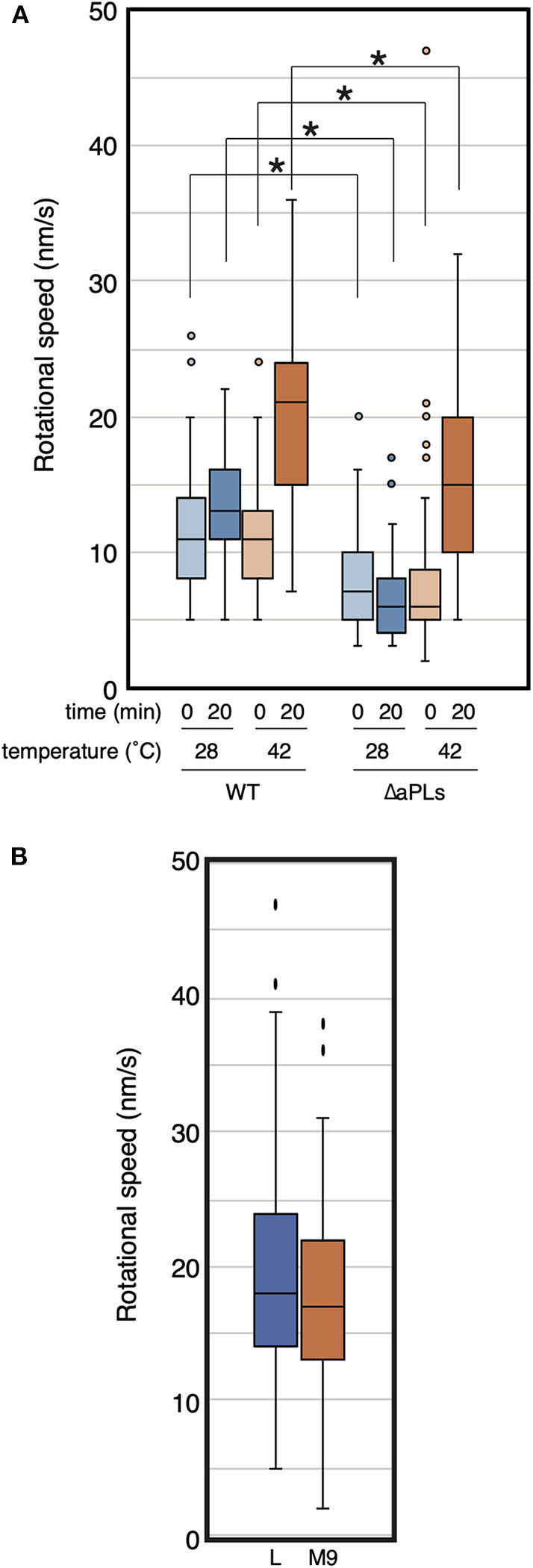
Rotational speed of MreB at different temperatures. **(A)** The distribution of the rotational speed (nm/s) of MreB in WT (RU1558) or ΔaPL (RU1527) cells is shown. The objective lens was heated at the indicated temperature for the indicated time. *P*-values were determined by the unpaired *t*-test. *P*-values < 0.05, shown by asterisks (*), were considered significantly different from each other. **(B)** The distribution of the rotational speed (nm/s) of MreB in WT (RU1558) grown in L or M9 medium at 37°C. The objective lens was heated at 37°C.

While we were examining MreB motion in the ΔaPLs mutant, we noticed that MreB rotated in the mutant at high temperatures. Thus, we analyzed the effect of temperature on MreB motion in ΔaPLs cells. ΔaPLs cells producing MreB-msfGFP^SW^ were grown to the log phase at 37°C, harvested, and mounted on an M9 agarose pad. We started the observation 5–10 min later, and the temperature was maintained at 28°C. We observed the motion of MreB at 0 and 20 min at 28°C (Figure 2A, Supplementary Movies 2A,B, and Supplementary Table 4). The average speeds of MreB at both time points were 8.0 ± 3.6 and 6.5 ± 2.7 nm/s, respectively. Then, we observed the motion of MreB 20 min after the objective lens was heated by a lens heater to 42°C. The speed of MreB was clearly increased (15.3 ± 6.7 nm/s). Because the increase in temperature should not increase the amount of aPLs in ΔaPLs cells (mutant that lacks the enzymes to synthesize aPLs), the results suggest a lack of aPLs; hence, different compositions of phospholipids are not a direct cause of the reduction in MreB motion. The rate of most biochemical processes generally increases with increasing temperature. In fact, it has been shown that the speed of MreB motion is dependent on temperature (van Teeffelen et al., 2011). We also examined the motion of MreB in WT cells when the temperature was increased and found that the speed of MreB motion increased 20 min after the objective lens was heated to 42°C (20.2 ± 6.3 nm/s) than that at 0 min (10.9 ± 3.7 nm/s) (Figure 2A and Supplementary Table 4). It is possible that as the temperature increased, the cell wall synthesis rate increased, and as a result, the rotation speed of MreB increased. Therefore, we examined if growth rate affects the rotational speed of MreB. RU1558 cells producing MreB-msfGFP^SW^ was grown in L or M9 medium at 37°C. Doubling times of cells grown in L and M9 were 28.0 ± 0.6 min and 68.6 ± 1.6 min, respectively. However, the rotational speeds of MreB in rich and poor medium were similar and they were not statistically different (*P*-values was 0.077) under our conditions (19.7 ± 8.7 nm/s for L medium and 17.6 ± 7.3 nm/s for M9 medium) (Figure 2B, Supplementary Movies 3A,B and Supplementary Table 4). This is consistent with previous reports (van Teeffelen et al., 2011; Billaudeau et al., 2017). This result suggest that decreased rate of peptidoglycan synthesis was not the major cause of decreased rotational speed of MreB.

### Effect of MurG Overproduction on MreB Motion in ΔaPLs

**Figure 3:**
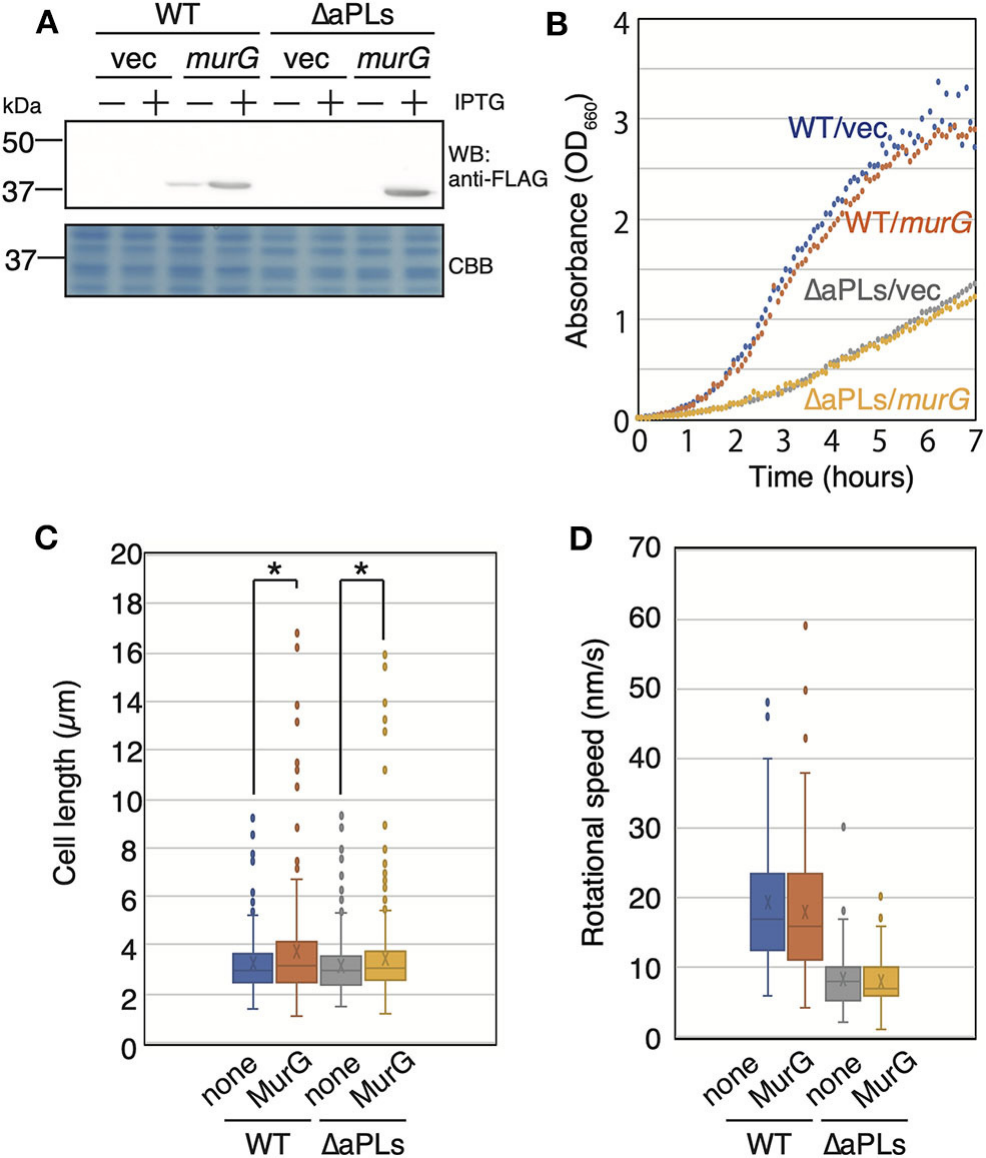
Effect of MurG on the rotation of MreB. **(A)** Protein levels of FLAG-tagged MurG (FLAG-MurG) in WT (MG1655) and ΔaPLs (RU835) cells grown in the absence or presence of 1 mM IPTG. Samples were subjected to SDS-polyacrylamide gel electrophoresis and immunoblotting using the anti-DDDDK (FLAG) antibody. As a loading control, a CBB-stained gel is also shown. **(B)** Growth of WT (MG1655) and ΔaPLs (RU835) cells carrying a vector or a plasmid encoding *flag-murG* at 37°C. **(C)** Cell length of WT (MG1655) and ΔaPLs (RU835) cells carrying a vector (none) or a plasmid encoding *flag-murG* (MurG) grown in the presence of 1 mM IPTG to the log phase at 37°C. *P*-values were determined by the unpaired *t*-test. *P* values < 0.05, shown by asterisks (*), were considered significantly different from each other. **(D)** The distribution of the rotational speed (nm/s) of MreB in WT (RU1558) or ΔaPL (RU1527) cells carrying a vector (none) or a plasmid encoding *flag-murG* (MurG).

The deletion of enzymes synthesizing cardiolipin (CL) has been shown to result in reduced activity of MurG, a GTase involved in lipid II biosynthesis, thereby causing abnormal cell shape in *Rhodobacter sphaeroides* (Lin et al., 2019). This shape deficiency was restored by MurG overproduction. On the other hand, *murG* expression is upregulated in *E. coli* lacking CL (van den Brink-van der Laan et al., 2003). However, aPLs mutant lacks phosphatidylglycerol (PG) in addition to CL. Thus, it is possible that activity of MurG in ΔaPLs is reduced as in *R. sphaeroides* ΔCL mutant. Therefore, we examined whether MreB rotation was restored in ΔaPLs cells by MurG overproduction (and consequently by restoring peptidoglycan synthesis). If MreB rotation was restored, it is plausible to conclude that decreased MreB rotation was caused by decreased peptidoglycan synthesis in ΔaPLs. First, we cloned FLAG-tagged *murG* in a plasmid and confirmed that MurG was produced in the presence of 1 mM IPTG (Figure 3A). Next, we examined whether the overproduction of MurG restored the slow-growth phenotype of ΔaPLs cells and found that MurG overproduction did not improve the slow growth of ΔaPLs (Figure 3B). Moreover, MurG overproduction did not promote the growth of WT cells (Figure 3B). However, both WT and ΔaPLs cells overproducing MurG (WT: 3.69 ± 2.06 μm, ΔaPLs: 3.46 ± 1.90 μm) were slightly but significantly longer than those carrying a vector plasmid (WT: 3.21 ± 1.14 μm, ΔaPLs: 3.18 ± 1.12 μm) and the numbers of longer WT and ΔaPL cells producing MurG were increased compared to those of control cells (Figure 3C), suggesting that peptidoglycan synthesis in both cells was promoted by MurG overproduction. Then, we observed the rotation of MreB in MurG-overproducing cells. As seen in Figure 3D, Supplementary Movies 4A,B, and Supplementary Table 4, MurG overproduction did not increase the rotational rate of MreB in WT (18.0 ± 10.0 nm/s) or ΔaPLs (7.9 ± 3.9 nm/s). These results suggest that the decrease in MreB rotation in ΔaPLs did not result from reduced peptidoglycan synthesis activity but rather from reduced membrane fluidity. In fact, membrane fluidity has been shown to be decreased in ΔaPLs cells (Nenninger et al., 2014). In that study, the authors measured the diffusion coefficient of BODIPY FL-C_12_ in WT and ΔaPLs cells and showed that it is decreased in ΔaPLs (0.66 ± 0.22 μm^2^/s) compared with that in WT (1.2 ± 0.3 μm^2^/s). Therefore, we hypothesized that the cause of the decrease in the motion of MreB in ΔaPLs cells was the decrease in the membrane fluidity. Thus, we next examined the effect of membrane fluidity on MreB motion.

### Effect of Membrane Fluidity on MreB Motion

The amount of unsaturated fatty acids is related to membrane fluidity (Marr and Ingraham, 1962). FabA and FabB are essential proteins to synthesize unsaturated fatty acids (Feng and Cronan, 2009). Moreover, it was recently shown that the depletion of FabB reduces membrane fluidity (Budin et al., 2018).

We first constructed a strain in which the expression of the *fabA* gene is controlled by arabinose to change the membrane fluidity under a constant temperature. The growth of the strain was dependent on arabinose (Supplementary Figure 2A), confirming that FabA is essential for viability. This result is consistent with previous reports that unsaturated fatty acids, which comprise at least 15–20% of total phospholipids, are required for growth (Cronan and Gelmann, 1973). Cells were grown in the presence of arabinose overnight, and the culture was diluted with fresh medium either in the presence (+FabA, shown by orange dots) or absence of arabinose (–FabA, shown by blue dots) (Supplementary Figure 2A). Then, we examined the cell shape of FabA-depleted cells in the presence and absence of arabinose at around 3 h (shown by a gray bar) (Supplementary Figure 2B). WT (MG1655) cells hardly exhibited any abnormal shapes such as bends (only in 1.7% of the cells, i.e., 9/553 cells). However, FabA-depleted cells grown in the absence of arabinose exhibited abnormal shapes such as bends in 29% of the cells (48/168 cells). We measured the length and width of FabA-depleted cells grown in the absence and presence of arabinose (Supplementary Figure 2C) and found that cell widths significantly increased when cells were grown in the absence of arabinose compared with those grown with arabinose, suggesting that the cell width was not well regulated, especially in cells grown without arabinose (Supplementary Figures 2B,C). These results indicate that FabA depletion affected the cell shape. It should be noted that FabA-depleted cells grown in the presence of 0.2% arabinose also showed abnormal shape in 19% of the cells (42/222 cells), suggesting that the quantity of FabA may not be optimized in the cells.

**Figure 4:**
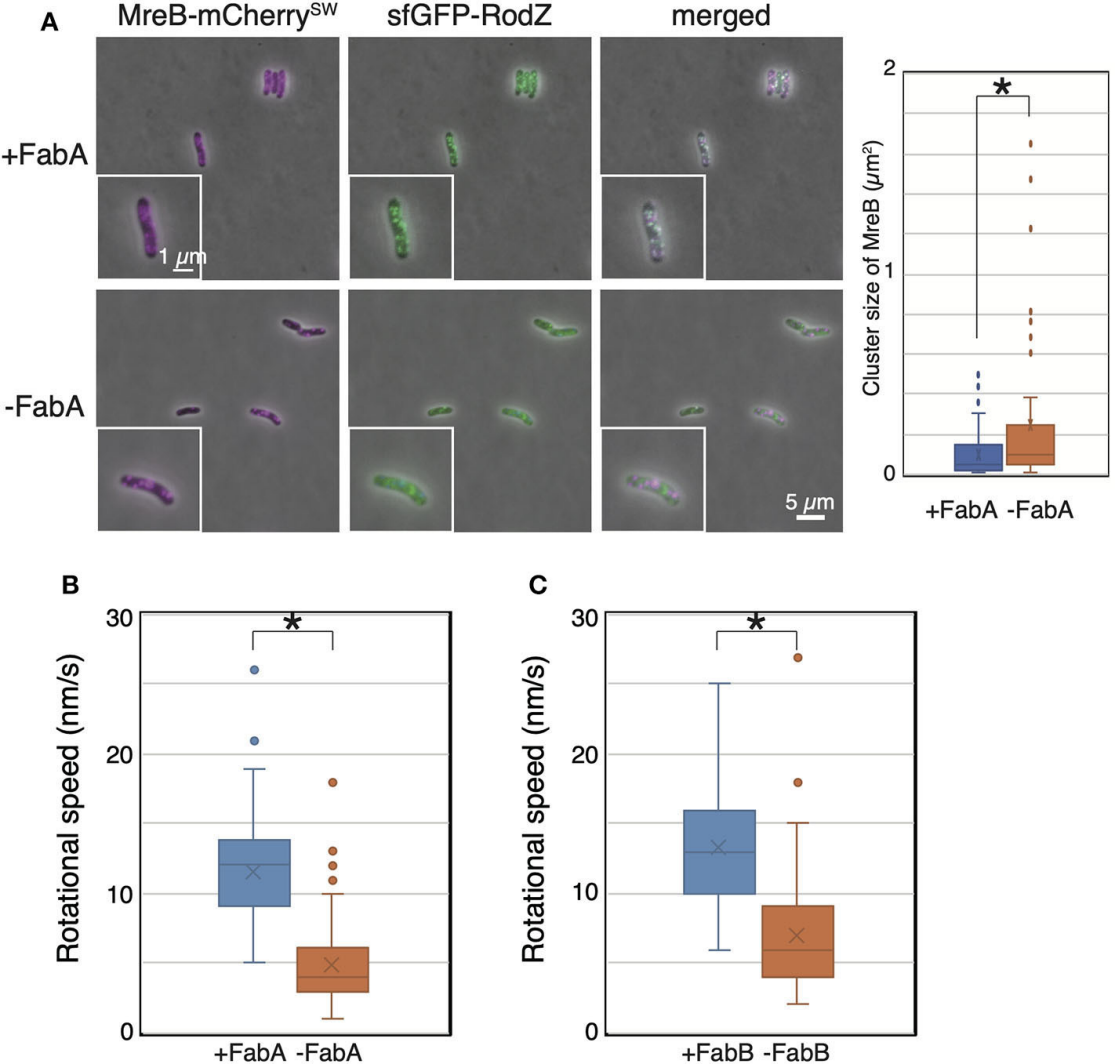
Localization and motion of MreB and RodZ in cells depleted of FabA or FabB. **(A)** Localization of MreB and RodZ in FabA-depleted cells in the presence (+FabA) and absence (–FabA) of arabinose. Merged images of phase-contrast and fluorescent images are shown. A magnified cell is shown in the inset. Scale bar: 5 μm. **(B)** The distribution of the rotational speed (nm/s) of MreB in FabA-depleted cells in the presence (+FabA) and absence (–FabA) of arabinose is shown. **(C)** The distribution of the rotational speed (nm/s) of MreB in FabB-depleted cells in the presence (+FabB) and absence (–FabB) of arabinose is shown. *P*-values were determined by the unpaired *t-*test. *P*-values < 0.05, shown by asterisks (*), were considered significantly different from each other.

Then, we observed the localization and dynamics of MreB-mCherry^SW^ and sfGFP-RodZ in the FabA-depleted strain (RU1504). MreB and RodZ formed clusters, as in WT cells, in the presence of arabinose (+FabA) (Figure 4A). In contrast, in cells grown in the absence of arabinose (–FabA), MreB formed clusters that were often abnormally large and appeared to be aggregates (Figure 4A). We found that MreB clusters in FabA-depleted cells were larger than those in +FabA cells (Figure 4A). In addition, some RodZ did not form clusters but was diffused in the cells (–FabA) compared with that in the cells grown with arabinose (+FabA) (Figure 4A). These results suggest that membrane fluidity affects the formation of MreB and RodZ clusters and hence, the formation of the Rod complex. We found that the motion of MreB in a FabA-depleted strain (RU1504) grown in the absence of arabinose (5.5 ± 3.0 nm/s) clearly decreased compared to that in the presence of arabinose (10.8 ± 4.0 nm/s) (Figure 4B, Supplementary Movies 5A,B, and Supplementary Table 4). These results suggest that FabA, and thus membrane fluidity, affect the localization of MreB and RodZ and their motion. However, it is possible that FabA, not membrane fluidity, somehow directly affects the localization and motion of MreB.

Thus, we constructed a FabB-depleted strain. FabB is also an essential protein and functions downstream of FabA in the synthesis pathway of unsaturated fatty acids. We examined the subcellular localization of MreB and RodZ in a FabB-depleted strain grown in the presence (+FabB) or absence (–FabB) of arabinose. As in FabA-depleted cells, some MreB and RodZ formed large clusters (indicated by white arrows), and MreB and RodZ did not colocalize (indicated by green and magenta arrows) in cells grown in the absence of arabinose (–FabB) (Supplementary Figure 3). Interestingly, there were cells in which MreB did not form clusters but RodZ did (shown by green arrows in Supplementary Figure 3) and vice versa (shown by magenta arrows in Supplementary Figure 3). These results combined with the results obtained by FabA-depleted cells suggest that membrane fluidity affects the formation of the Rod complex. We observed the motion of MreB-msfGFP^SW^ in the FabB-depleted strain RU1822 (MG1655 *mreB-msfGFP*^*SW*^ Δ*fabB::kan*/pBAD-*fabB*). In the presence of arabinose (+FabB), MreB formed clusters, as in WT, and moved around the short axis of the cell (11.9 ± 4.0 nm/s) (Figure 4C, Supplementary Movie 5C, and Supplementary Table 4). However, in the absence of arabinose (–FabB), MreB formed clusters, as in WT cells, but the rotational speed of MreB was decreased (6.9 ± 3.9 nm/s) (Figure 4C, Supplementary Movie 5D, and Supplementary Table 4). These results indicate that depletion of either FabA or FabB resulted in decreased motility of MreB, and that membrane fluidity plays a key role in MreB motion.

## Discussion

In this study, we found that speeds of the rotational motion of MreB and RodZ significantly decreased in cells lacking aPLs, indicating that aPLs affect the motion of MreB and RodZ (Rod complex). We showed that the interaction between MreB and RodZ in ΔaPLs was comparable with that in WT cells, suggesting that the composition of phospholipids did not affect the interaction between MreB and RodZ.

The rotational speed of MreB has been shown to be dependent on temperature (van Teeffelen et al., 2011). We also showed that the rotational speed increased when the temperature was increased in WT and ΔaPLs cells, indicating that the decreased speed of MreB in ΔaPLs cells did not result from the lack of aPLs. The rotation of the Rod complex is coupled with the activity of peptidoglycan synthesis and is dependent on temperature (Domínguez-Escobar et al., 2011; Garner et al., 2011; van Teeffelen et al., 2011). Membrane fluidity is also dependent on temperature (Zhang and Rock, 2008). Therefore, the cause of the influence of temperature on rotational speed might be the change in peptidoglycan synthesis activity or membrane fluidity.

Inhibition of peptidoglycan synthesis suppresses the dynamics of the Rod complex, indicating that the movement of the Rod complex is coupled with peptidoglycan synthesis (Domínguez-Escobar et al., 2011; Garner et al., 2011; van Teeffelen et al., 2011). Therefore, it is possible that the decrease in the membrane fluidity did not directly cause the inhibition of the dynamics of the Rod complex but decreased the peptidoglycan synthetic activity, and consequently, the motion of the Rod complex was inhibited. To clarify this, we conducted two experiments; (1) MreB rotation in cells grown in rich and poor medium at the same temperature and (2) MreB rotation in cells overproducing MurG which should promote peptidoglycan synthesis. However, the rotational speed of MreB was similar whether cells were grown in rich or poor medium. This is consistent with previous reports (van Teeffelen et al., 2011; Billaudeau et al., 2017). Deletion of CL has been shown to result in reduced activity of MurG, which is a GTase involved in peptidoglycan synthesis, and therefore, it caused abnormal shape in *Rhodobacter sphaeroides* (Lin et al., 2019), while it was shown that expression of *murG* was upregulated in *E. coli* ΔCL cells (van den Brink-van der Laan et al., 2003). This abnormal shape in *R. sphaeroides* was restored to a normal rod shape by inducing *murG* expression because MurG overproduction improved peptidoglycan synthesis. This expression level of *murG* did not affect doubling time of ΔCL cells but possibly increased peptidoglycan synthesis. Therefore, to distinguish whether the reduced motion of MreB in ΔaPLs was due to reduced peptidoglycan synthesis or reduced membrane fluidity, MurG was overproduced in ΔaPLs, and we examined its effect on MreB motion. We found that the overproduction of MurG resulted in the elongation of cells but did not affect MreB rotation. These results suggest that decreased peptidoglycan synthesis was not the major cause of reduced rotational speed of MreB. However, elongation of the cells overproducing MurG was very subtle. Therefore, it is possible that overproduction of MurG did not significantly increase peptidoglycan synthesis but inhibited cell division, and hence, rational speed of MreB was not increased. In fact, it is difficult to distinguish following possibilities; (1) overproduction of MurG enhanced peptidoglycan synthesis but did not affect the rotational speed of MreB, and (2) overproduction of MurG inhibited cell division and did not affect the rotational speed of MreB. Further experiments will be needed to clarify the possibilities by examination of peptidoglycan synthesis in ΔaPLs overproducing MurG.

Nevertheless, we hypothesized that membrane fluidity is one of the important factors for the rotation of MreB because it has been shown that phosphatidylglycerol exhibits relatively higher fluidity (Pluschke and Overath, 1981), the membrane fluidity of ΔaPLs cells is lower than that of WT cells (Nenninger et al., 2014), and MreB influences membrane fluidity (Strahl et al., 2014). To decrease membrane fluidity by means other than decreasing the temperature, we depleted FabA or FabB, which are essential in the synthesis of unsaturated fatty acids determining membrane fluidity (Marr and Ingraham, 1962). When either FabA or FabB was depleted, some cells showed aberrant shapes, such as a bend or elongation. In addition, in FabA- or FabB-depleted cells, MreB apparently formed clusters, and some MreB formed larger clusters similar to aggregates. However, some MreB did not completely colocalize with RodZ in those cells. Thus, membrane fluidity affects the formation and motion of the Rod complex, and therefore, FabA- or FabB-depleted cells show an aberrant cell shape. During the preparation of this manuscript, a manuscript by Gohrbandt et al. was uploaded in bioRxiv, in which a clear defect of divisome assembly was observed upon depletion of unsaturated fatty acids in the *E. coli fabA* (Ts) mutant, while MreB still formed clusters in the mutant as in WT cells (Gohrbandt et al., 2019). However, they did not study the localization of other proteins in the Rod complex, such as RodZ. Thus, membrane fluidity affects protein complex formation during cell division and elongation.

We showed the inhibition of MreB motion in ΔaPLs cells and FabA-/FabB-depleted cells and aberrant formation of Rod complexes only in FabA-/FabB-depleted cells. It is possible that the reduced membrane fluidity independently affected the motion and formation of the Rod complex. As ΔaPLs cells can still produce FabA and FabB, the decrease in the membrane fluidity of aPLs cells may not be as great as that of FabA-/FabB-depleted cells. Thus, there is a smaller effect on the formation of the Rod complex, but the impact on complex dynamics may be significant in ΔaPLs cells. While this manuscript was under review, it was reported that membrane fluidity controls MreB motion in *Bacillus subtilis* (Zielińska et al., 2020). Thus, membrane fluidity controls the motion of the Rod complex in both gram-positive and gram-negative bacteria. Therefore, the mechanism would be widely conserved in bacterial cells. In *B. subtilis*, it has been reported that the motion of Mbl (an MreB-like protein) is inhibited by the depletion of components of the Rod complex such as RodZ and RodA, suggesting that the correct formation of the Rod complex is critical for its dynamics. In *E. coli*, the Δ*rodZ* strain (in which the morphology changes into a round shape) has been found to reduce MreB rotation (Morgenstein et al., 2015). However, when MreB carries the S14A mutation that suppresses the morphological abnormality of Δ*rodZ* cells, the motility of MreB (MreB^S14A^) is restored (Hussain et al., 2018). This is probably because MreB^S14A^ is able to correctly form the Rod complex without RodZ, so MreB^S14A^ can rotate. This idea is consistent with the results that the formation of the Rod complex is perturbed and the motion of the Rod complex is inhibited in FabA-/FabB-depleted cells.

As membrane fluidity is modulated by external conditions such as changes in temperature and pH, cells may regulate peptidoglycan synthesis by changing membrane fluidity to survive in various harsh environments.

## Data Availability Statement

The raw data supporting the conclusions of this article will be made available by the authors, without undue reservation.

## Author Contributions

KK, FK, and DS made contributions to the design of the study, the acquisition, analysis, and interpretations of the data. KK and DS made contributions to writing of the manuscript. All authors contributed to the article and approved the submitted version.

## Conflict of Interest

The authors declare that the research was conducted in the absence of any commercial or financial relationships that could be construed as a potential conflict of interest.
