# Supplementary material for: Alteration of Membrane Fluidity or Phospholipid Composition Perturbs Rotation of MreB Complexes in Escherichia coli
Source: Front Mol Biosci. 2020 Nov 24;7:582660. doi: 10.3389/fmolb.2020.582660 (PMC7719821; doi:10.3389/fmolb.2020.582660)
Supplement: Supplementary file 1 [file Data_Sheet_1.PDF]

Supplementary Material

Alteration of membrane fluidity or phospholipid composition perturbs rotation of MreB complexes in *Escherichia coli*

Keisuke Kurita, Fumiya Kato, and Daisuke Shiomi

1 Supplementary Methods

1.1 Strain construction

The primers used for strain construction are listed in Supplementary Table S2.

RU1126 (*ΔrlmN::kan sfGFP-rodZ*) To insert Kan^R, which is flanked by FRT sites that can be used to excise the cassette with pCP20, into the *rlmN* gene, which is upstream of *rodZ*, pKD4 was amplified using primers 1292 and 1293. The PCR product was introduced into strain RU383 carrying pKD46 by electroporation, yielding RU1126 (*ΔrlmN::kan sfGFP-rodZ*).

RU1181 (*ΔrlmN sfGFP-rodZ*) and **RU1178 (*ΔrlmN* ΔaPLs *sfGFP-rodZ*)** A P1 lysate prepared form RU1126 was used to transduce *ΔrlmN::kan sfGFP-rodZ* into MG1655 and RU835 to yield RU1139 (*ΔrlmN::kan sfGFP-rodZ*) and RU1140 (*ΔrlmN::kan* ΔaPLs *sfGFP-rodZ*). These strains were transformed with the plasmid pCP20 using selection for Amp^R at 30°C. The resulting transformants were incubated at 42°C in the absence of Amp, and colonies that grew were screened for the Amp^S and Kan^S phenotype at 37°C. The final strains were designated RU1181 (*ΔrlmN sfGFP-rodZ*) and RU1178 (*ΔrlmN* ΔaPLs *sfGFP-rodZ*).

RU1184 (*ΔcsrD::kan mreB-mCherry^{SW} ΔrlmN sfGFP-rodZ*) and **RU1185 (*ΔcsrD::kan* ΔaPLs *mreB-mCherry^{SW} ΔrlmN sfGFP-rodZ*)** A P1 lysate prepared from RU856 (*ΔcsrD::kan mreB-mCherry^{SW}*) was used to transduce *ΔcsrD::kan mreB-mCherry^{SW}* into RU1181 (*ΔrlmN sfGFP-rodZ*) and RU1178 (*ΔrlmN* ΔaPLs *sfGFP-rodZ*) to yield RU1184 (*ΔcsrD::kan mreB-mCherry^{SW} ΔrlmN sfGFP-rodZ*) and RU1185 (*ΔcsrD::kan* ΔaPLs *mreB-mCherry^{SW} ΔrlmN sfGFP-rodZ*).

RU1557, RU1558 (*mreB-msfGFP^{SW}*) and **RU1524, RU1527 (ΔaPLs *mreB-msfGFP^{SW}*)** A P1 lysate prepared form NO50 (Ouzounov et al., 2016; Ursell et al., 2014) in which *kan^R* was inserted between *csrD* and *mreB* genes was used to transduce *kan^R-mreB-msfGFP^{SW}* into MG1655 and RU835 to yield RU1557 (*mreB-msfGFP^{SW}*) and RU1524 (ΔaPLs *mreB-msfGFP^{SW}*). These strains were transformed with the plasmid pCP20 using selection for Amp^R at 30°C. The resulting transformants were incubated at 42°C in the absence of Amp, and colonies that grew were screened for the Amp^S and Kan^S phenotype at 37°C. The final strains were designated RU1558 (*mreB-msfGFP^{SW}*) and RU1527 (ΔaPLs *mreB-msfGFP^{SW}*).

RU1364 (*ΔcsrD::cat mreB-mCherry^{SW} ΔyhdE*), **RU1446 (*ΔcsrD mreB-mCherry^{SW}*)**, **RU1447 (*ΔrlmN::kan sfGFP-rodZ ΔcsrD mreB-mCherry^{S}*)** and **RU1448 (*ΔrlmN sfGFP-rodZ ΔcsrD mreB-*

mCherry^{SW}) A P1 lysate prepared from RU305 (*ΔcsrD::cat mreB-mCherry^{SW} ΔyhdE*) was used to transduce *ΔcsrD::cat mreB-mCherry^{SW} ΔyhdE* into MG1655 to yield RU1364 (*ΔcsrD::cat mreB-mCherry^{SW} ΔyhdE*). The *cat* cassette was flanked by FRT sites that can be used to excise the cassette with pCP20. RU1364 (*ΔcsrD::cat mreB-mCherry^{SW}*) was transformed with the plasmid pCP20 using selection for Amp^R at 30°C. The resulting transformants were incubated at 42°C in the absence of Amp, and colonies that grew were screened for the Amp^S and Cm^S phenotype at 37°C, yielding RU1446 (*ΔcsrD mreB-mCherry^{SW}*). A P1 lysate prepared form RU1126 was used to transduce *ΔrlmN::kan sfGFP-rodZ* into RU1146, yielding RU1447 (*ΔcsrD mreB-mCherry^{SW} ΔrlmN::kan sfGFP-rodZ*). and RU1447 (*ΔcsrD mreB-mCherry^{SW} ΔrlmN::kan sfGFP-rodZ*) were transformed with the plasmid pCP20 using selection for Amp^R at 30°C. The resulting transformants were incubated at 42°C in the absence of Amp, and colonies that grew were screened for the Amp^S and Cm^S phenotype at 37°C, yielding RU1448 (*ΔrlmN sfGFP-rodZ ΔcsrD mreB-mCherry^{SW}*).

RU1504 (*ΔrlmN sfGFP-rodZ ΔcsrD mreB-mCherry^{SW} ΔfabA::kan* / pRU1501), **RU1816 (*ΔrlmN sfGFP-rodZ ΔcsrD mreB-mCherry^{SW} ΔfabB::kan* / pRU1758)**, **RU1822 (*mreB-msfGFP^{SW} ΔfabB::kan* / pRU1758),** and **RU1875 (*ΔfabB::kan* / pRU1758)** To insert Kan^R into the *fabA* or *fabB* gene, pKD4 was amplified using primers 1805 and 1806 for *fabA* deletion and 1966 and 1967 for *fabB* deletion. The PCR products were introduced into strain RU1448 (*ΔrlmN sfGFP-rodZ ΔcsrD mreB-mCherry^{SW}*), RU1558 (*ΔcsrD mreB-msfGFP^{SW}*), MG1655 (WT) carrying pKD46 and pRU1501 (*fabA*) or pRU1758 (*fabB*) by electroporation, yielding RU1504 (*ΔrlmN sfGFP-rodZ ΔcsrD mreB-mCherry^{SW} ΔfabA::kan* / pRU1501), RU1816 (*ΔrlmN sfGFP-rodZ ΔcsrD mreB-mCherry^{SW} ΔfabB::kan* / pRU1758), RU1822 (*mreB-msfGFP^{SW} ΔfabB::kan* / pRU1758), and RU1875 (*ΔfabB::kan* / pRU1758).

RU1271 (MG1655 *Δcya*) and **RU1333 (RU835 *Δcya*)** To construct strains for the bacterial two-hybrid assay, a P1 lysate prepared from JW3787 (BW25113 *Δcya*) was used to transduce *Δcya::kan* into MG1655 and RU835. The resulting strains were transformed with the plasmid pCP20 to remove *kan^R* using selection for Amp^R at 30°C. The resulting transformants were incubated at 42°C in the absence of Amp, and colonies that grew were screened for the Amp^S and Kan^S phenotype at 37°C, yielding RU1271 (MG1655 *Δcya*) and RU1333 (RU835 *Δcya*).

1. 2 Plasmid construction

The plasmids used in this study are listed in Supplementary Table S3. For the construction of all plasmids, KOD-plus-Neo DNA polymerase (TOYOBO, Osaka, Japan) was used for PCR experiments and T4 ligase (New England BioLabs, MA, USA) was used for ligations. Primers used for plasmid construction are listed in Supplementary Table S2.

pRU1276 ClaI-HindIII fragment of pBAD33 was replaced with the corresponding fragment of pBAD24, yielding pRU1276.

pRU1565 PstI-ScaI fragment of pDSW208 containing MCS-*gfp* was replaced with the corresponding fragment containing MCS (without gfp), yielding pRU1565.

pRU2007 PstI-ScaI fragment of pRU1565 was replaced with the corresponding fragment of pDSW208F, yielding pRU2007 (pDSW208F no GFP).

pRU2009 The *murG* gene was amplified using genomic DNA of MG1655 as template and primers 2123 and 2128. The PCR product was cut with SacI and XbaI and the fragment was cloned into the corresponding site of pRU2007, yielding pRU2009.

pRU1501 (pRU1276-*fabA*) and **pRU1758 (pRU1276-*fabB*)** The *fabA* and *fabB* gene were amplified using genomic DNA of MG1655 as template and primers 1803 and 1804 for *fabA* or 1964 and 1965 for *fabB*. The PCR products were cut with XbaI and HindIII for *fabA* or SmaI and PstI for *fabB*, and the fragments were cloned into the corresponding site of pRU1276, yielding pRU1501 (*fabA*) and pRU1758 (*fabB*).

pRU1917 (pKNT25-*mreB-T25*^{SW}) and **pRU1918 (pUT18-*mreB-T18*^{SW})** As 1st PCR, *mreB*$^{1\text{-}228}$, *mreB*$^{229\text{-}347}$ and T25 or T18 amplified using genomic DNA of MG1655 (*mreB*$^{1\text{-}228}$), pKNT25 (*T25*) or pUT18 (*T18*) as template and primers 545 and 2084 (*mreB*$^{1\text{-}228}$ for *mreB-T25*^{SW}: PCR product #1), 545 and 2080 (*mreB*$^{1\text{-}228}$ for *mreB-T18*^{SW} : PCR product #4), 2085 and 546 (*mreB*$^{229\text{-}347}$ for *mreB-T25*^{SW} : PCR product #2), 2081 and 546 (*mreB*$^{229\text{-}347}$ for *mreB-T18*^{SW} : PCR product #5), 2083 and 2086 (*T25* for *mreB-T25*^{SW} : PCR product #3), or 2079 and 2082 (T18 for *mreB-T18*^{SW} : PCR product #6). As 2nd PCR, *mreB*$^{1\text{-}228}$*-T25* and *mreB*$^{1\text{-}228}$*-T18* were amplified using mixtures of PCR products #1 /#3 and #4 / #6 as templates and primers 545/2086 (*mreB*$^{1\text{-}228}$*-T25*: PCR product #7) and 545/2082 (*mreB*$^{1\text{-}228}$*-T18* : PCR product #8). As 3rd PCR, *mreB-T25*^{SW} and *mreB-T18*^{SW} were amplified using mixtures of PCR products #2 / #7 and #5 / #8 as templates and primers 545/546 for both constructs. The PCR products were cut with BamHI and EcoRI, and the fragments were cloned into the corresponding site of pKNT25 or pUT18 to yield pRU1917 (pKNT25-*mreB-T25*^{SW}) and pRU1918 (pUT18-*mreB-T18*^{SW}).

2 Supplementary Figures and Tables

2.1 Supplementary Figures

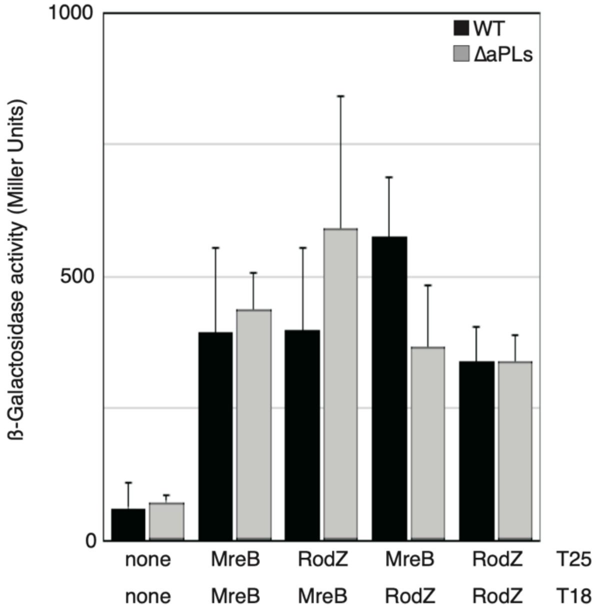

Supplementary Figure S1. Interactions between RodZ and MreB as determined using bacterial two-hybrid (BACTH) assays. Interactions between MreB and RodZ (R) or self-interactions were quantified by measuring the β-galactosidase activity (Miller units) of WT or ΔaPLs cells carrying plasmids producing the indicated proteins fused to either the T25 or T18 fragment of adenylate cyclase. Average Miller units and standard deviations are shown (*n* = 3). Black and gray bars indicate WT and ΔaPLs, respectively.

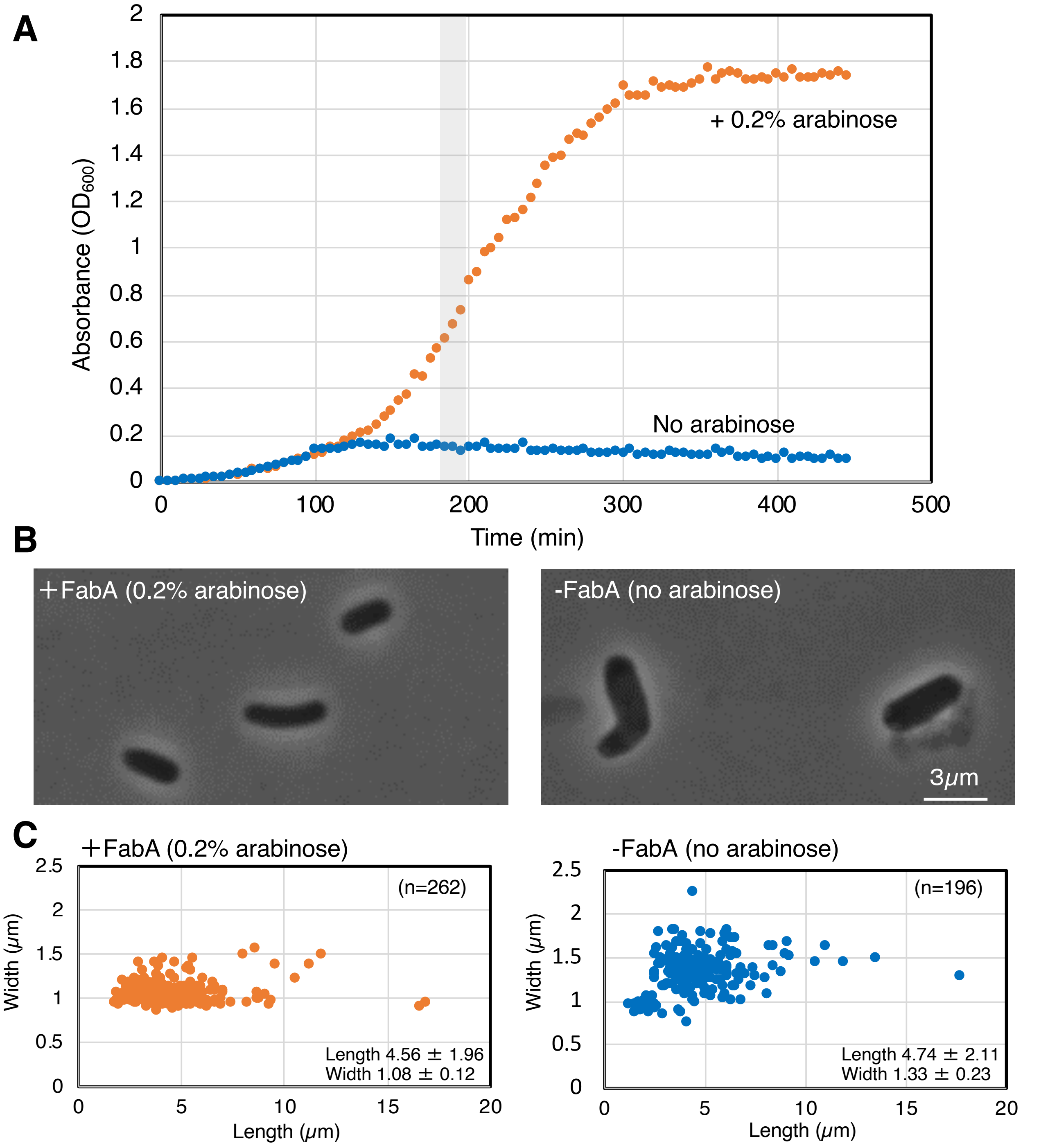

Supplementary Figure S2. Growth and cell shape of FabA-depletion **cells.** (A) Growth of FabA-depletion cells grown in the presence (orange dots) and absence (blue dots) of arabinose. Arabinose was added at time 0. (B) Phase-contrast images of FabA-depletion cells grown in the presence and absence of arabinose. Scale bar: 3 μm. (C) The distributions of cell lengths and widths obtained from measurements of almost 200 cells grown in the presence and absence of arabinose are shown.

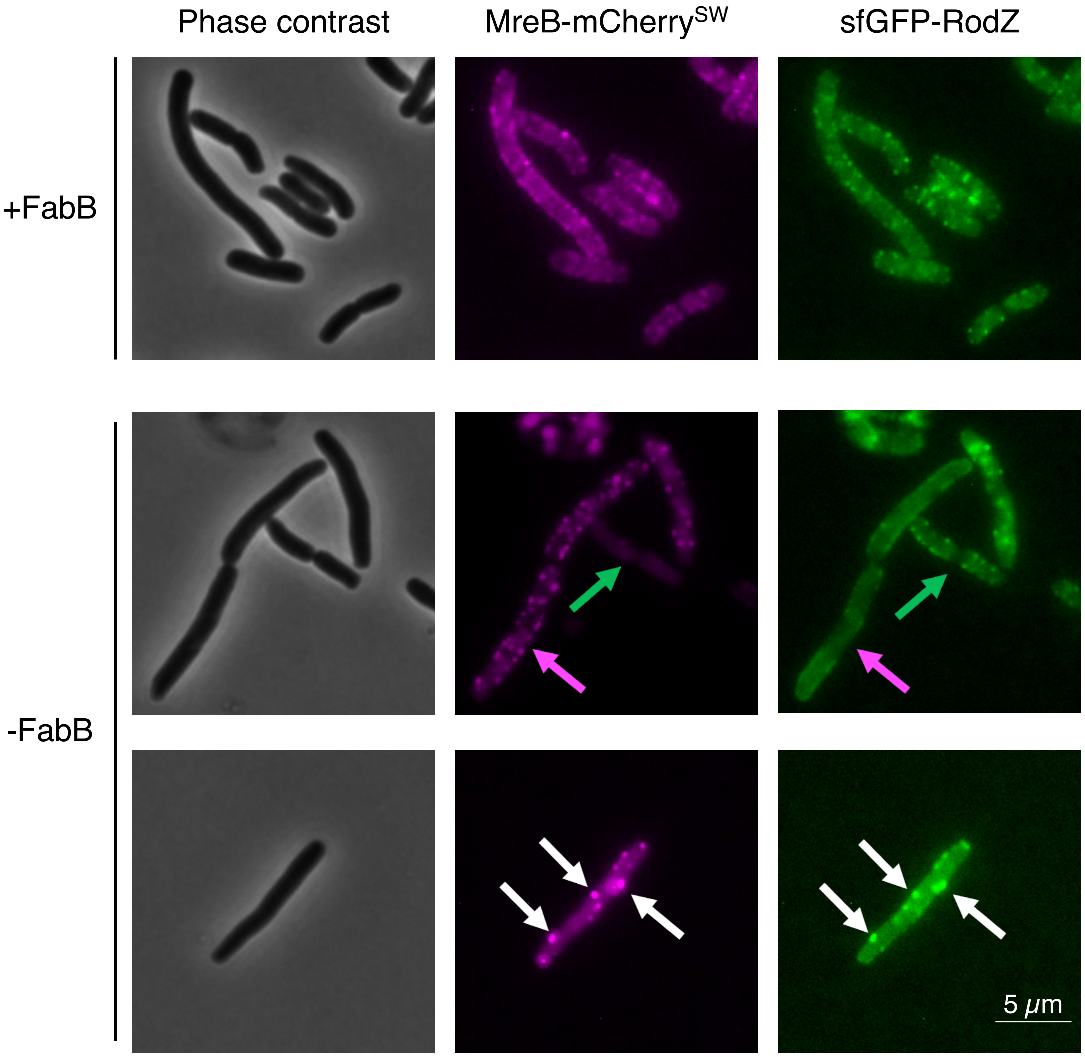

Supplementary Figure S3. Localization of MreB and RodZ in FabB-depleted cells. FabB-depleted cells grown in the presence (+FabB) and absence (-FabB) of arabinose. Phase-contrast and fluorescent images are shown. White arrows indicate the colocalization of MreB and RodZ. Green and magenta arrows indicate cells in which MreB did not form clusters but RodZ did and vice versa, respectively. Scale bar: 5 μm

2.2 Supplementary Tables

Supplementary Table S1. Strains used in this study

Strain	Relevant genotypes	References
MG1655	WT	(Guyer *et al*., 1981)
NO50	MG1655 *mreB-msfGFP*^{sw} *ΔcsrD::kan*	(Ouzounov *et al*., 2016)
RU305	BW25113 *mreB-mCherry*SW *ΔyhdE ΔcsrD::cat*	(Yoshii et al., 2019)
RU383	BW25113 sfGFP-*rodZ*	(Ikebe et al., 2018)
RU835	MG1655 derivatives *ΔpgsA ΔclsABC ΔymdB lpp2 Δara714 rcsF::mini-Tn10*$_{cam}$ (same as BKT29 but *kan*^{S})	(Tan *et al*., 2012) (Kawazura *et al*., 2017)
RU856	MG1655 *mreB-mCherry*^{SW} *ΔcsrD::kan ΔyhdE*	(Kawazura *et al*., 2017)
RU857	RU835 *mreB-mCherry*^{SW} *ΔcsrD::kan ΔyhdE*	(Kawazura *et al*., 2017)
RU1126	BW25113 sfGFP-*rodZ ΔrlmN::kan*	This study
RU1139	MG1655 sfGFP-*rodZ ΔrlmN::kan*	This study
RU1140	RU835 sfGFP-*rodZ ΔrlmN::kan*	This study
RU1178	RU835 sfGFP-*rodZ ΔrlmN*	This study
RU1181	MG1655 sfGFP-*rodZ ΔrlmN*	This study
RU1184	MG1655 *mreB-mcherry*^{sw} *ΔcsrD::kan ΔyhdE sfGFP-RodZ ΔrlmN*	This study
RU1185	RU835 *mreB-mcherry*^{sw} *ΔcsrD::kan ΔyhdE sfGFP-RodZ ΔrlmN*	This study
JW3778	BW25113 *ΔcyaA::kan*	(Baba *et al*., 2006)

RU1271	MG1655 Δ*cyaA*	This study
RU1333	RU835Δ*cyaA*	This study
RU1364	MG1655 *mreB-mCherry*^{sw} Δ*csrD::cat* Δ*yhdE*	This study
RU1446	MG1655 *mreB-mCherry*^{sw} Δ*csrD* Δ*yhdE*	This study
RU1447	MG1655 *mreB-mCherry*^{sw} Δ*csrD* Δ*yhdE* *sfGFP-rodZ* Δ*rlmN::kan*	This study
RU1448	MG1655 *mreB-mCherry*^{sw} Δ*csrD* Δ*yhdE* *sfGFP-rodZ* Δ*rlmN*	This study
RU1504	MG1655 *mreB-mcherry*^{sw} Δ*csrD* Δ*yhdE* *sfGFP-RodZ* Δ*rlmN* Δ*fabA::kan* / pBAD33MCS3-*fabA*	This study
RU1524	RU835 *mreB-msfGFP*^{sw} *Kan*^{R}	This study
RU1527	RU835 *mreB-msfGFP*^{sw} *Kan*^{S}	This study
RU1557	MG1655 *mreB-msfGFP*^{sw} *Kan*^{R}	This study
RU1558	MG1655 *mreB-msfGFP*^{sw} *Kan*^{S}	This study
RU1816	MG1655 *mreB-mCherry*^{SW} Δ*csrD* Δ*yhdE* *sfGFP-rodZ* Δ*rlmN* Δ*fabB*::*kan* / pBAD33MCS3-*fabB*	This study
RU1822	MG1655 *mreB-msfGFP*^{sw} Δ*fabB*::*kan* / pBAD33MCS3-*fabB*	This study
RU1875	MG1655 Δ*fabB*::*kan* / pBAD33MCS3-*fabB*	This study

Supplementary Table S2. Primers used in this study

Primer (number)	Sequence
mreB-f(BamHI)noATG (546)	GCGGATCCGTTGAAAAAATTTCGTGGCATG
mreB-r (EcoRI) (547)	GCGAATTCTTACTCTTCGCTGAACAGGTCG
fabA-f(XbaI) (1803)	GCTCTAGAGATGGTAGATAAACGCGAATC
fabA-r(HindIII) (1804)	GCAAGCTTTCAGAAGGCAGACGTATCCT
fabA-H1P1-f (1805)	GTGTTAGCTATCCTGCGTGCTTCAATAAAATAAGGCTTACA GAGAACATGGTGTAGGCTGGAGCTGCTTC
fabA-H2P2-r (1806)	TCGCCTTTTGATACTCTGTCTGATTATAATCAGAAGGCAGA CGTATCCTGCATATGAATATCCTCCTTA
fabB-f(smaI) (1964)	GCCCCGGGATGAAACGTGCAGTGATTAC
fabB-r(pstI) (1965)	GCCTGCAGTTAATCTTTCAGCTTGCGCA
fabB-H1P1-f (1966)	ATTGTGCATTCGAAACTTACTCTATGTGCGACTTACAGAGG TATTGAATGGTGTAGGCTGGAGCTGCTTC
fabB-H2P2-r (1967)	CTGGCGCGTCTACTCCGACCTACTGCGAATTAATCTTTCAG CTTGCGCATCATATGAATATCCTCCTTA
mreB-cyaT18-f (2079)	GGTTCGGCTTATCCGGGCGATAGCGGCTCGAGCGCCGCCA GCGAGGCCACGGG
mreB-cyaT18-r (2080)	CCCGTGGCCTCGCTGGCGGCGCTCGAGCCGCTATCGCCCG GATAAGCCGAACC
cyaT18-mreB-f (2081)	CGCTGGGCGCAGTGGAACGCTCTGGCGCGCCTGGCGAAGT CCGTGAAATCGAAGTTC
cyaT18-mreB-r (2082)	GAACTTCGATTTCACGGACTTCGCCAGGCGCGCCAGAGCG TTCCACTGCGCCCAGCG
mreB-cyaT25-f (2083)	GGTTCGGCTTATCCGGGCGATAGCGGCTCGAGCCAGCAAT CGCATCAGGCTGG

mreB-cyaT25-r (2084)	CCAGCCTGATGCGATTGCTGGCTCGAGCCGCTATCGCCCG GATAAGCCGAACC
cyaT25-mreB-f (2085)	CTGGCGCGCACGCGGCGGGCCTCTGGCGCGCCTGGCGAAG TCCGTGAAATCGAAGTTC
cyaT25-mreB-r (2086)	GAACTTCGATTTCACGGACTTCGCCAGGCGCGCCAGAGGC CCGCCGCGTGCGCGCCAG
murG-f(SacI) (2128)	GCGAGCTCAGTGGTCAAGGAAAGCGATTAATG
murG-r(XbaI) (2123)	GCTCTAGATTACGCCCGGGCAACCCGGC

Supplementary Table S3. Plasmids used in this study

Plasmid	Relevant genotype	References
pKD4	Kan^R flanked by FRT sites, R6K ori, Amp^R	(Datsenko and Wanner, 2000)
pKD46	Lambda Red recombinase, oriR101, *repA*^{ts}, Amp^R	(Datsenko and Wanner, 2000)
pCP20	yeast Flp recombinase gene, λcI857, λP_R Rep^{ts} Cm^R, Amp^R	(Cherepanov and Wackernagel, 1995)
pBAD24	P_{BAD} promoter followed by MCS3, pBR322 ori, Amp^R	(Guzman et al., 1995)
pBAD33	P_{BAD} promoter followed by MCS2*, p15A ori, Cm^R	(Guzman et al., 1995)
pRU1276	pBAD33 but carrying MCS3 (derived from pBAD24), p15A ori, Cm^R	This study
pRU1501	pRU1276-*fabA*, p15A ori, Cm^R	This study
pRU1758	pRU1276-*fabB*, p15A ori, Cm^R	This study
pDS1266	*rodZ* in pUT18C, Amp^R	(Yoshii *et al*., 2019)
pDS1271	*rodZ* in pKT25, Kan^R	(Yoshii *et al*., 2019)
pKNT25	P_{lac}::T25 (T25 tag for C-terminal fusion), p15A ori, Kan^R	(Karimova *et al*., 2001)
pRU1917	pKNT25-*mreB-T25*^{SW}, p15A ori, Kan^R	This study
pUT18	P_{lac}::T18 (T18 tag for C-terminal fusion), ColE1 ori, Amp^R	(Karimova *et al*., 2001)
pRU1918	pUT18-*mreB-T18*^{SW}, ColE1 ori, Amp^R	This study
pDSW207	weakened P_{trc} promoter, *gfp*-MCS, pBR322 ori, Amp^R	(Weiss *et al*., 1999)

pDSW208	weakened P_{trc} promoter, MCS-*gfp*, pBR322 ori, Amp^R	(Weiss *et al*., 1999)
pDSW208F	FLAG tag inserted between EcoRI and SacI site of pDSW208	Lab stock<br><br>(Shiomi and Margolin, 2007)
pRU1565	pDSW208 derivative, weakened P_{trc} promoter, no gfp, pBR322 ori, Amp^R	This study
pRU2007	FLAG tag inserted between EcoRI and SacI site of pRU1565	This study
pRU2009	*FLAG-murG* in pRU2007	This study

Supplementary Table S4. Summary of rotational speeds of MreB and RodZ under various conditions

Strain	Protein	Speed (nm/sec)	Conditions	Related fig.
RU1184 (WT)	MreB-mCherry^{SW}	14.0 ± 3.8	28˚C	Fig. 1
	sfGFP-RodZ	14.1 ± 4.0	28˚C	Fig. 1
RU1185 (ΔaPLs)	MreB-mCherry^{SW}	7.1 ± 4.1	28˚C	Fig. 1
	sfGFP-RodZ	8.5 ± 3.8	28˚C	Fig. 1
RU1558 (WT)	MreB-msfGFP^{SW}	11.2 ± 3.6	28˚C (t=0 min)	Fig. 2
		13.5 ± 3.8	28˚C (t=20 min)	Fig. 2
		10.9 ± 3.7	28˚C (t=0 min)	Fig. 2
		20.2 ± 6.3	42˚C (t=20 min)	Fig. 2
RU1527 (ΔaPLs)	MreB-msfGFP^{SW}	8.0 ± 3.6	28˚C (t=0 min)	Fig. 2
		6.5 ± 2.7	28˚C (t=20 min)	Fig. 2
		7.8 ± 5.7	28˚C (t=0 min)	Fig. 2
		15.3 ± 6.7	42˚C (t=20 min)	Fig. 2
RU1558 (WT)	MreB-msfGFP^{SW}	19.7 ± 8.7	grown in L medium at 37˚C	Fig. 2
		17.6 ± 7.3	grown in M9 medium at 37˚C	Fig.2
RU1558 (WT)/ pRU2007 (vector)	MreB-msfGFP^{SW}	19.1 ± 9.1	NA	Fig. 3
RU1558 (WT)/ pRU2009 (*murG*)	MreB-msfGFP^{SW}	18.0 ± 10.0	MurG overproduction	Fig. 3
RU1527 (ΔaPLs) /pRU2007 (vector)	MreB-msfGFP^{SW}	8.2 ± 4.5	NA	Fig. 3
RU1527 (ΔaPLs) /pRU2009 (*murG*)	MreB-msfGFP^{SW}	7.9 ± 3.9	MurG overproduction	Fig. 3
RU1504	MreB-mCherry^{SW}	5.5 ± 3.0	no arabinose (-FabA)	Fig. 4
(FabA-depletion strain)		10.8 ± 4.0	with arabinose (+FabA)	Fig. 4
RU1822	MreB-msfGFP^{SW}	6.9 ± 3.9	no arabinose (-FabB)	Fig. 4
(FabB-depletion strain)		11.9 ± 4.0	with arabinose (+FabB)	Fig. 4

Supplementary References

Baba, T., Ara, T., Hasegawa, M., Takai, Y., Okumura, Y., Baba, M., et al. (2006). Construction of Escherichia coli K-12 in-frame, single-gene knockout mutants: the Keio collection. *Mol. Syst. Biol.* 2, 2006.0008. doi:10.1038/msb4100050.

Cherepanov, P. P., and Wackernagel, W. (1995). Gene disruption in Escherichia coli: TcR and KmR cassettes with the option of Flp-catalyzed excision of the antibiotic-resistance determinant. *Gene* 158, 9–14. doi:10.1016/0378-1119(95)00193-a.

Datsenko, K. A., and Wanner, B. L. (2000). One-step inactivation of chromosomal genes in Escherichia coli K-12 using PCR products. *Proc. Natl. Acad. Sci. U.S.A.* 97, 6640–6645. doi:10.1073/pnas.120163297.

Guzman, L. M., Belin, D., Carson, M. J., and Beckwith, J. (1995). Tight regulation, modulation, and high-level expression by vectors containing the arabinose PBAD promoter. *J. Bacteriol.* 177, 4121–4130. doi:10.1128/jb.177.14.4121-4130.1995.

Ikebe, R., Kuwabara, Y., Chikada, T., Niki, H., and Shiomi, D. (2018). The periplasmic disordered domain of RodZ promotes its self-interaction in Escherichia coli. *Genes Cells* 23, 307–317. doi:10.1111/gtc.12572.

Karimova, G., Ullmann, A., and Ladant, D. (2001). Protein-protein interaction between Bacillus stearothermophilus tyrosyl-tRNA synthetase subdomains revealed by a bacterial two-hybrid system. *J. Mol. Microbiol. Biotechnol.* 3, 73–82.

Kawazura, T., Matsumoto, K., Kojima, K., Kato, F., Kanai, T., Niki, H., et al. (2017). Exclusion of assembled MreB by anionic phospholipids at cell poles confers cell polarity for bidirectional growth. *Mol. Microbiol.* 104, 472–486. doi:10.1111/mmi.13639.

Ouzounov, N., Nguyen, J. P., Bratton, B. P., Jacobowitz, D., Gitai, Z., and Shaevitz, J. W. (2016). MreB Orientation Correlates with Cell Diameter in Escherichia coli. *Biophys. J.* 111, 1035–1043. doi:10.1016/j.bpj.2016.07.017.

Shiomi, D., and Margolin, W. (2007). The C-terminal domain of MinC inhibits assembly of the Z ring in Escherichia coli. *J. Bacteriol.* 189, 236–243. doi:10.1128/JB.00666-06.

Ursell, T. S., Nguyen, J., Monds, R. D., Colavin, A., Billings, G., Ouzounov, N., et al. (2014). Rod-like bacterial shape is maintained by feedback between cell curvature and cytoskeletal localization. *Proc. Natl. Acad. Sci. U.S.A.* 111, E1025–34. doi:10.1073/pnas.1317174111.

Weiss, D. S., Chen, J. C., Ghigo, J. M., Boyd, D., and Beckwith, J. (1999). Localization of FtsI (PBP3) to the septal ring requires its membrane anchor, the Z ring, FtsA, FtsQ, and FtsL. *J. Bacteriol.* 181, 508–520.

Yoshii, Y., Niki, H., and Shiomi, D. (2019). Division-site localization of RodZ is required for efficient Z ring formation in Escherichia coli. *Mol. Microbiol.* 111, 1229–1244. doi:10.1111/mmi.14217.
